# Highly Dispersible Buckled Nanospring Carbon Nanotubes for Polymer Nano Composites

**DOI:** 10.1038/s41598-018-23172-1

**Published:** 2018-03-19

**Authors:** Y. J. Lee, S. R. Ham, J. H. Kim, T. H. Yoo, S. R. Kim, Y. T. Lee, D. K Hwang, B. Angadi, W. S. Seo, B. K. Ju, W. K. Choi

**Affiliations:** 1 Center for Opto-Electronic Materials and Devices, Post-Silicon Semiconductor Institute, Korea Institute of Science and Technology (KIST), Hwarangro 14 gil 5, Sungbuk Gu, Seoul 02792 Korea; 2 Department of Electronic, Electrical, and Computer Engineering, College of Engineering, Korea University, 145 Anam-ro, Seongbuk-gu, Seoul 02841 Korea; 3 Division of Nano & Information Technology, KIST School, Korea University of Science and Technology (KUST), Seoul, 02792 Republic of Korea; 4 Department of Polymer Engineering, Korea National University of Transportation, 50 Daehak-ro, Chungju-si, Chungbuk 27469 Korea; 5 Department of Physics, Bangalore University, Bangalore, 560 056 India; 6 Korea Institute of Ceramic Engineering and Technology, 101, Soho-ro, Jinju-si, Gyeongsangnam-do 52851 Korea

## Abstract

We propose the unique structure of highly dispersible single-walled carbon nanotubes (SWCNTs) in various solvents and polymers using the ZnO nano particle template. Buckled nanospring-shaped carbon nanotubes (NS-CNTs) were synthesized by a chemical reaction of ZnO nanoparticles with acid-treated SWCNTs and then dissolving ZnO through chemical etching. The unique structure of distorted hexagonal NS-CNTs encircled around ZnO nanoparticles was formed by the bending of SWCNTs caused by the agglomeration of chemically adsorbed Zn(OH)_2_, which is further crystallized as the polycrystalline ZnO inner core. The highly dispersible NS-CNTs could be incorporated in the poly[(vinylidenefluoride-co-trifluoroethylene] [P(VDF-TrFE)] copolymer, one of widely studied ferro- and piezo-electric polymer, up to the value of 15 wt% as nanofillers. The relative dielectric constant (*K*) of polymer nanocomposite, at 1 kHz, was greatly enhanced from 12.7 to the value of 62.5 at 11 wt% of NS-CNTs, corresponding to a 492% increase compared to that of pristine P(VDF-TrFE) with only a small dielectric loss tangent (*D*) of 0.1.

## Introduction

CNTs possess excellent properties and have been actively researched for applications in various fields such as nanoelectronic devices, nanobalance, energy harvesting, and flexible conducting electrodes^1–5^. While CNTs have extraordinary properties, their low dispersion in various solvents or polymer matrix (not taking advantage of any nanoscale effects) hinders their applicability for the projected devices. In order to enhance the dispersion of CNTs, many efforts have been reported in literatures^6–10^. For instance, some methods involve a block copolymer consisting of conjugated polymers and mechanical dispersion, and the chemical surface modification method^11^. Another method involves the modification of shape of CNTs (circles or curved), but this method disrupts each connection there by degrading the properties of CNTs^12–15^. Therefore, it is necessary to find a new method to disperse CNTs without the degradation of properties, low yield or poor stability. Since the transformation of CNTs themselves is difficult, the use of CNTs at the metal oxide surface is recommended to change CNTs shape and hence their dispersibility. The studies on synthesis of carbon based materials with metal oxide have been well reported^16,17^, particularly the chemical exfoliation of graphene using ZnO^18,19^. But these hybrid metal oxides cannot make the unique structure like buckled nanospring. The NS-CNTs were blended with P(VDF-TrFE) and the electrical properties were evaluated to confirm the high dispersibility. There have been several reports on the enhanced properties of P(VDF-TrFE) based composites with the incorporation of metal nanoparticles^20,21^, high dielectric ceramic nanopowders^22,23^, hybrid oxide with metal particles^24,25^, hybrid polymers with oxides^26^, and CNTs^27^. However, P(VDF-TrFE) based high dielectric constant composites using carbon based organic materials have not been reported yet. Although the dispersibility of the linear CNTsis increased using the reported chemical and mechanical methods, loss value increases because they make networking in the dielectric matrix due to their linear shape. But when CNTs are fabricated into the buckled nanospring shape close to the sphere, there will be much more interfacial area. As mentioned above, this causes a lot more interfacial polarization that result in the enhancement of the dielectric constant. In this paper, we report a unique synthesis of NS-CNTs through the synthesis of ZnO-CNTs nanoparticles by using the thermal hydrolysis method and then removing ZnO by acid treatment. In order to demonstrate the dispersibility and electrical properties of buckled NS-CNTs, we have fabricated the nanocomposite of buckled NS-CNTs in P(VDF-TrFE) polymer and have studied their dielectric properties.

## Results and Discussion

**Figure 1:**
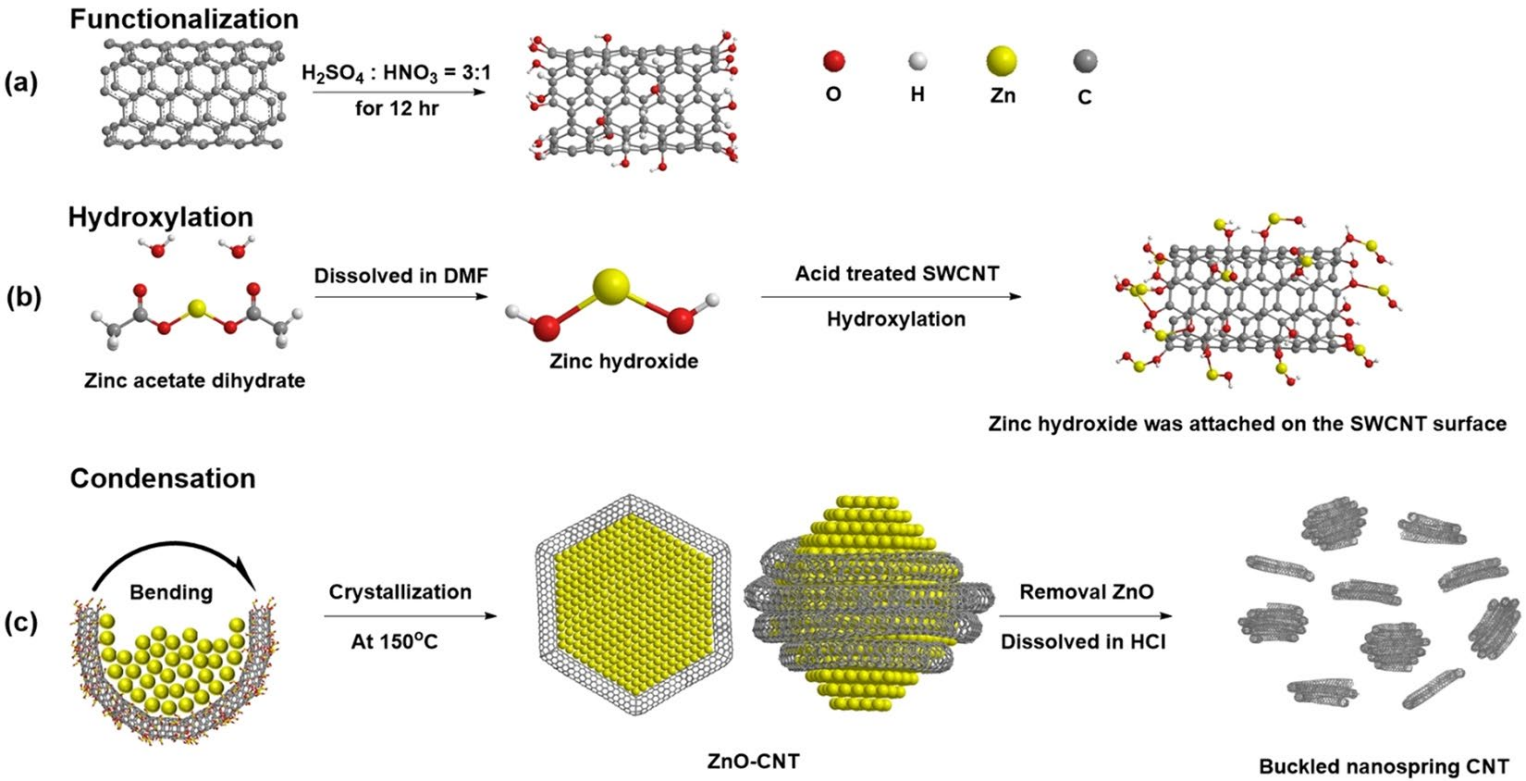
Chemical synthesis process for the buckled NS-CNTs. (**a**) Schematic showing the acid treatment of SWCNTs for functionalization. (**b**) Synthesis of Zn(OH)_2_ from zinc acetate dihydrate using the hydroxylation method. Synthesized Zn(OH)_2_ attached to functional groups on acid treated SWCNTs. (**c**) SWCNTs were bent by growth and aggregation of attached Zn(OH)_2_. For the synthesis of buckled NS-CNTs, ZnO-CNTs were dissolved in HCl to remove ZnO.

**Figure 2:**
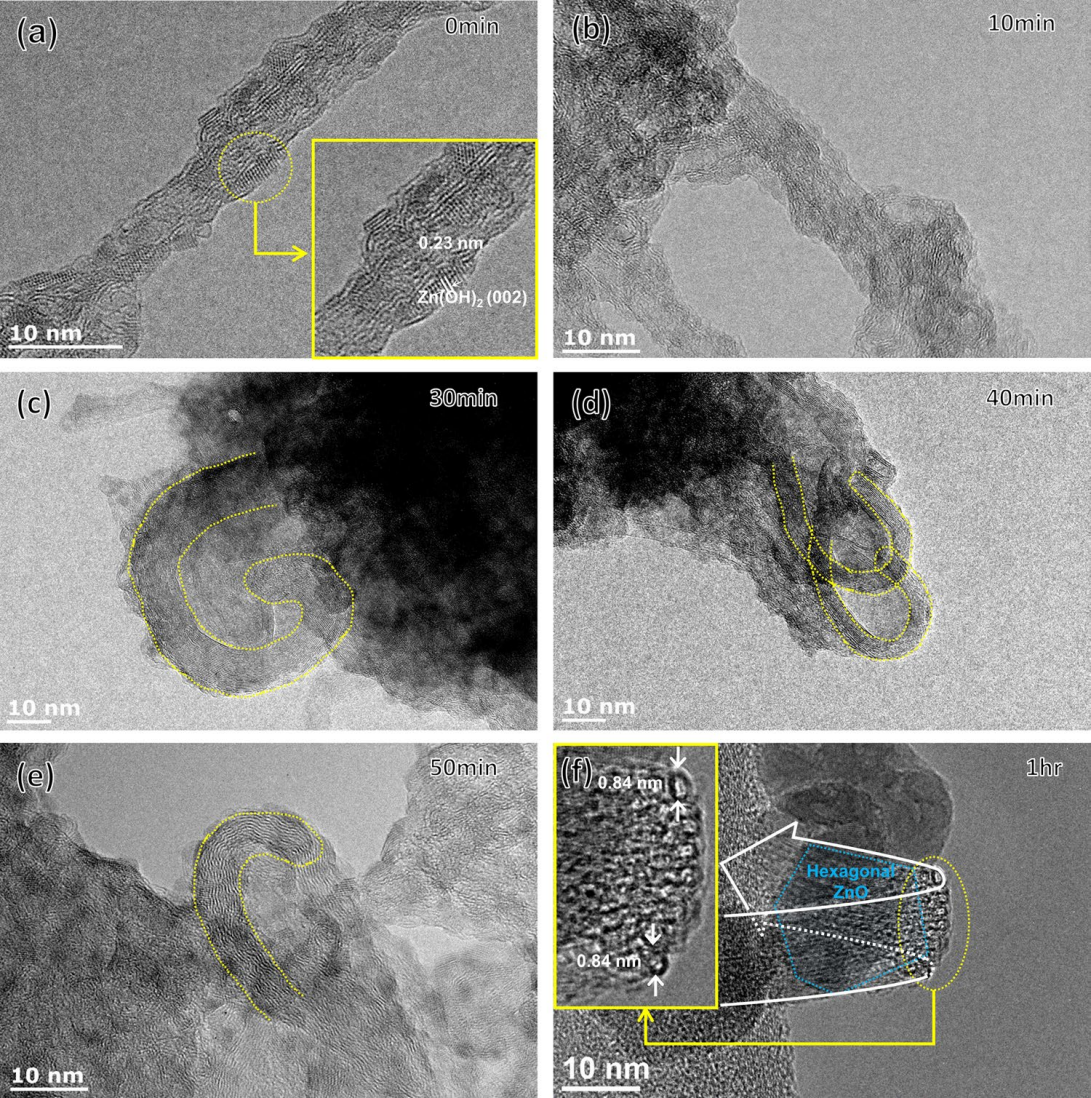
HR TEM image of ZnO-SWCNTs complex synthesized at various reaction time: (**a**) and (**b**) Zn(OH)_2_ was attached to SWCNTs surface. (**c**,**d** and **e**) CNTs were bent as seeds of Zn(OH)_2_ grew and aggregated. (**f**) CNTs turns (wide white arrow) wrapped ZnO nanoparticle (blue hexagon) and buckled NS-CNTs (dotted-yellow circle).

**Figure 3:**
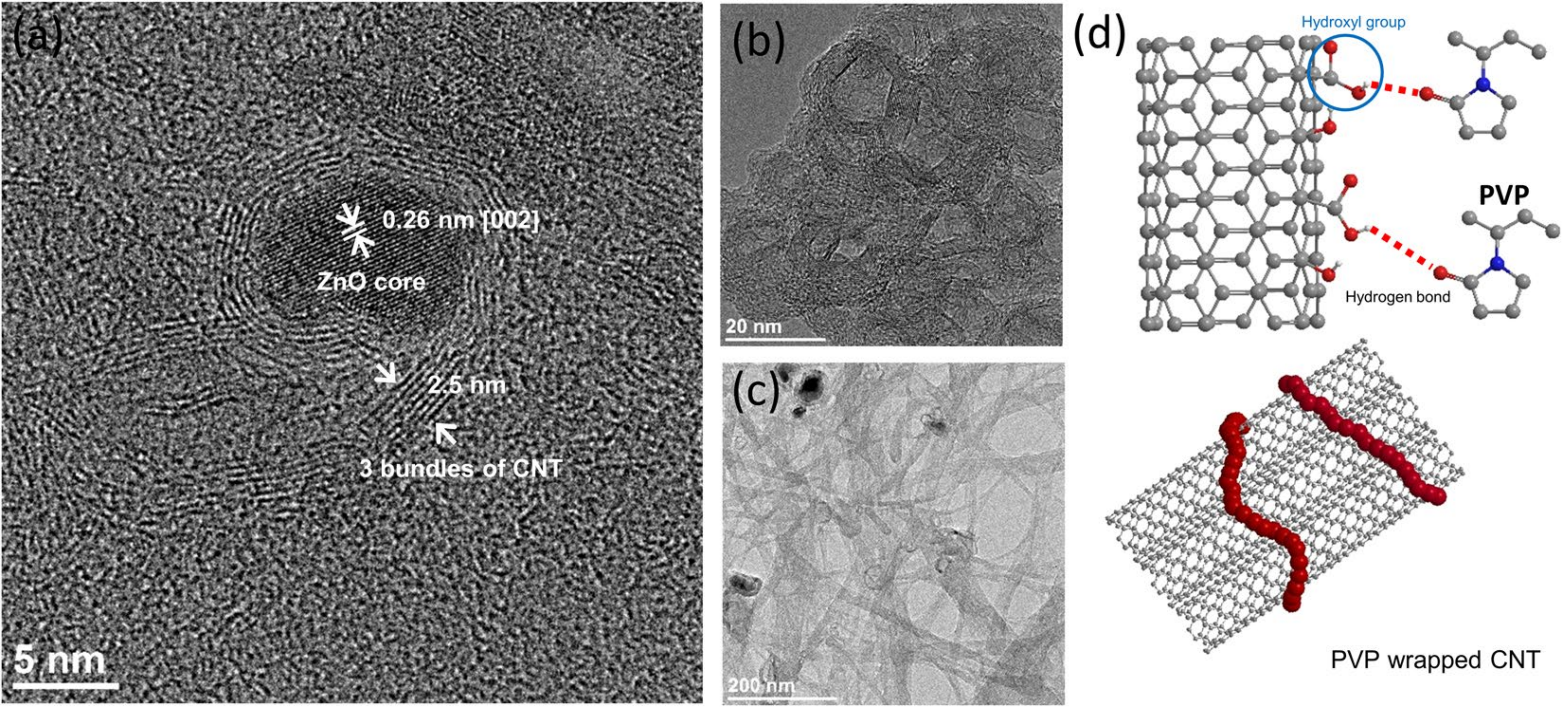
The role of PVP and the schematic diagram. (**a**) HR TEM images of ZnO-CNTs hybrid structure. (**b**) NS-CNTs after dissolving the core ZnO in HCl, sonication and sieving with AAO filter and (**c**) after sonication without AAO filter. (**d**) Schematic illustration of hydroxyl group on the surface of SWCNTs reacting with PVP and CNT turns bound with PVP (red color).

The sequence of process involved in the chemical synthesis of NS-CNTs is schematically shown in Fig. 1. The functionalization of as-procured SWCNTs through acid treatment introduces hydroxyl, epoxy, carbonyl, and carboxyl groups on the surface of SWCNTs (Fig. 1a). During the formation of the ZnO-CNTs hybrid structure, a phase of Zn(OH)_2_ gets attached on the surface of SWCNTs after ca. 10 min of reaction time through the chemical reaction between Zn(OH)_2_ and the functional groups on SWCNTs (Figs 1b and 2a,b). With an increase of a reaction time longer than 20 min, SWCNTs start to bend and distort locally due to the aggregation of Zn(OH)_2_ phase (Figs 1c and 2c,d,e). A further increase of reaction time beyond 1 h converts Zn(OH)_2_ into ZnO through dehydration. Subsequently, the number of ZnO nanoparticles (NPs) on the SWCNTs surfaces increases through agglomeration and nucleation. This results in the ZnO NPs (sizes up to 10 nm) crystallizing into a hexagonal structure (Fig. 2f). The ZnO inner core, wrapped by the SWCNTs, is clearly shown by the observed hexagonal structure with an interplanar spacing of 0.26 nm for [002] crystalline plane. The long axis of ZnO is estimated to be around 10 nm. The thickness of the SWCNTs surrounding the inner ZnO is about 2.5 nm, a length equal to the diameter of the three turns of SWCNTs (Fig. 3a). Also, the length of 6.75 nm corresponds to the diameter of the eight turns of SWCNTs. (Figure S1). The synthetic process of ZnO nanoparticles by reacting Zinc acetate dihydrate with acid treated SWCNTs was carefully investigated through analyzing x-ray diffraction patterns as the evolution of reaction time. (Figure S2). The Zinc acetate (Zn(OAc)_2_) becomes Zinc hydroxide (Zn(OH)_2_) that is occurred when water or hydroxyl group is encountered, that is a reversible/irreversible reaction. As a result, the peak intensity of Zinc acetate appeared at around 2θ = 11° may vary with reaction time^28^. As the reaction time increases up to 60 min, the XRD peak of SWCNTs existing around 2θ = 25° tends to decrease because Zinc hydroxide adheres to the surface of SWCNTs. Any XRD peaks related to ZnO crystalline structure could not be observed before the reaction time 50 min, but start to appear after 1 hr reaction time. (This is also clearly observed in TEM images as shown in Fig. 2f.) The relative composition of materials in ZnO-CNTs is analyzed using thermogravimetric analysis (TGA) (Figure S3). For the synthesis of NS-CNTs, the ZnO-CNTs were dissolved in hydrochloric acid (HCl) and subjected to sonication. Then, the product was sieved using an anodic aluminum oxide (AAO) membrane filter (Fig. 3b). As shown clearly in Fig. 3b, the nanosized and ring- shaped SWCNTs, called buckled NS-CNTs, were obtained. Figure S4(a) shows the XRD patterns of both ZnO-CNTs and ZnO-CNTs/PVP obtained after 5 hr reaction time respectively. The XRD peaks were observed at 2θ = 32.01°, 34.69°, 36.49°, 47.72°, 56.69°, 63.07°, 66.55°, 68.12°, 69.36°, and 77.11° and could be well assigned to (100), (002), (101), (102), (110), (103), (200), (112), (201), and (202) corresponding to crystalline structure of wurtzite hexagonal ZnO bulk. From the inset (an enlarged XRD feature between 2θ = 15° and 40°), the peak appeared at 2θ = 25° with low intensity is assigned to (002) of SWCNTs. Although ZnO-CNTs are wrapped by PVP, there is no difference in the XRD patterns of both ZnO-CNTs and ZnO-CNTs/PVP. Figure S4(b) shows the XRD patterns for neat SWCNTs, acid treated SWCNTs and NS-CNTs/PVP. The peaks appeared at 2θ = 25.5° and 43.59° correspond to (002) and (100) crystal planes of SWCNTS. In addition, the XRD peak at 2θ = 40° is found and corresponds to impurity of Pt catalyst, which was widely used for synthesizing neat SWCNTs by CVD. This peak is also observed in the acid treated SWCNTs. This Pt catalyst remains as the residual the TGA data (black line) as shown in the below Figure S5. Whereas no characteristic XRD peaks of any impurities like Pt in the NS-CNTs/PVP were observed and no residual was detected in the TGA data (red line) as shown in Figure S5, suggesting that synthesized NS-CNTs/PVP in this study have high quality. The size of the buckled NS-CNTs is about 20–30 nm and the shape resembles a distorted hexagon closely related to the crystalline structure of the ZnO NPs. There have been some reports on the formation of ring shaped CNTs using several approaches^12–15^, but the formation of buckled NS-CNTs with a size smaller than 30 nm has not been reported yet. Because of less controllability of the adsorbed Zn(OH)_2_ on the SWCNTs with different length, diameters and different amounts of functional groups, at this moment it still remains to be solved how the number of layers, radius, and distorted shapes of SWCNTs are well controlled. When making a ring-shaped CNT without using a template, there is a proposed method of producing CNT tangles by adjusting the gas velocity and frequency of acoustic waves^29^.

Nonetheless, the production yield of NS-CNTs from this method was very low (<4%). In addition, when the final product was not sieved with AAO filter after ultrasonication, most of the buckled NS-CNTs reverted back to their original linear appearance, as shown in Fig. 3c. This result implied that a lot of buckled NS-CNTs are formed via weak bonding. In order to further improve the production yield of buckled NS-CNTs, these weakly bonded buckled SWCNTs were treated with polyvinylpyrrolidone (PVP) polymer. It is expected that there will be strong hydrogen bonding between hydroxyl group (-OH) on SWCNTs and oxygen in PVP ((C_6_H_9_NO)_n_) and that this bonding may play a role as a strong wrapper causing effective binding between SWCNTs, as shown in Fig. 3d. This binding presumably allows the SWCNTs to keep their ring shape without breaking. From TGA graph, the relative weight content of PVP in buckled spring CNTs with PVP was revealed to the value of 23% (Figure S5). By adopting the PVP wrapper, the productivity of buckled NS-CNTs increased up to 98%.

**Figure 4:**
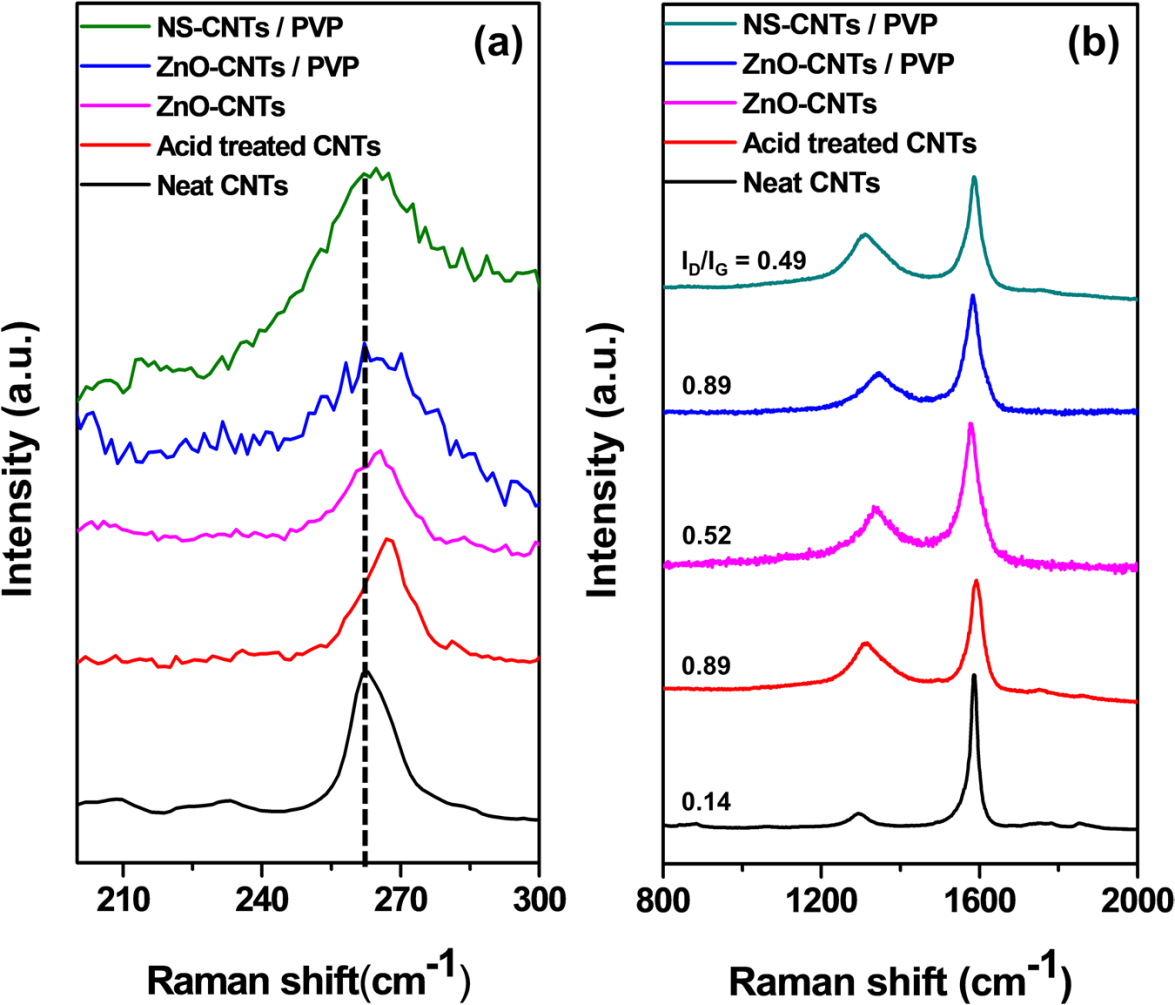
Raman spectra show different kinds of CNTs. (**a**) RBM region (**b**) high frequency region. Carbon nanotube diameter (d_t_) was determined by using d_t_(nm) = 224(cm^−1^)/ω(cm^−1^).

Raman spectroscopy is a basic tool for the measurement of CNT structure and quality. Figure 4 shows the Raman spectra of the pristine, acid treated SWCNTs, ZnO-CNT/PVPs and NS-CNTs. The radial breathing mode (RBM) intensity is related to SWCNTs diameter. The tube diameters about 0.84 nm yield identical results using the equation written in caption of Fig. 4. The RBM showed blue Raman shift was shown after functionalization because the functional groups on the wall of SWCNTs surface disrupt the radial vibration. A peak appear at 1300∼1400 cm^−1^ is called D-band indicating the degree of disorder and another peak at 1500∼1600 cm^−1^ is called G-band indicating tangential vibrational mode of graphitic materials. The intensity ratio I_D_/I_G_ is closely related to the structural quality of CNT. This ratio of 0.14 for neat SWCNTs increased after acid treatment up to 0.89, which is due to the induced oxygen-contained functional groups on the SWCNTs surface. Thereafter, zinc acetate must react with a hydroxyl group on the SWCNTs surface to form zinc hydroxide and Zn(OH)_2_ adhered to the surface of SWCNTs was crystallized into ZnO by a thermal hydrolysis. In the meantime, the hydroxyl groups, a defect in the SWCNTs, reacted with zinc acetate disappears and thus the ratio is reduced to 0.52 by the decrease of the D-band. Thereafter, in the process of chemical interaction of PVP with ZnO-CNTs, the adhered PVP influences the vibrational mode of D-band of the SWCNTs to increase the ratio up to 0.89. Removal of ZnO through acid treatment lowers the I_D_/I_G_ ratio down to 0.49 by reduction of oxygen defects of (Zn-O)-SWCNTs, which is not as low as the neat CNT because the PVP still binds to the CNTs. The calculation method for the I_D_/I_G_ ratio of all materials is shown in Figure S6.

**Figure 5:**
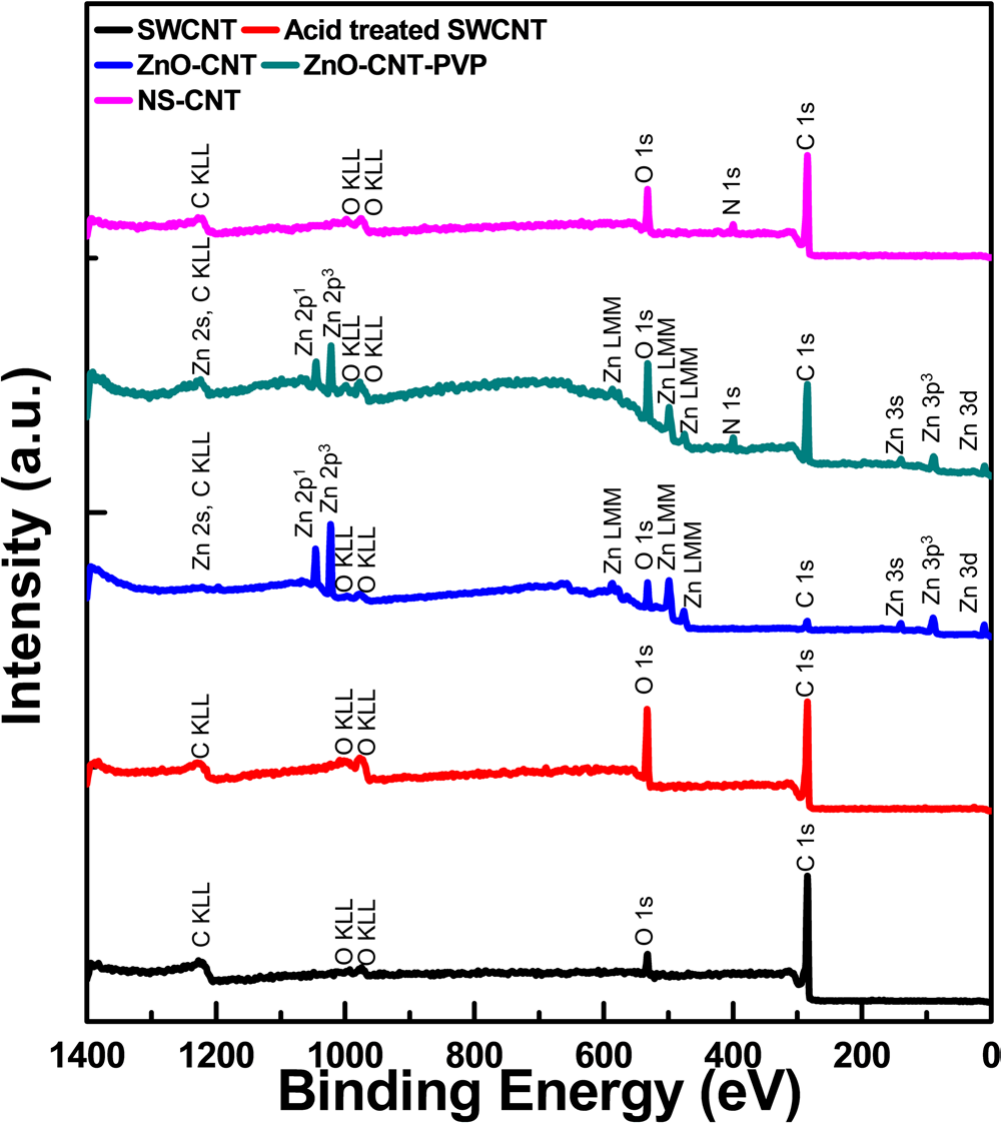
The XPS survey spectra of SWCNTs (black), acid treated SWCNTs (red), ZnO-CNTs (blue), ZnO-CNTs-PVP (green) and NS-CNTs (pink).

**Figure 6:**
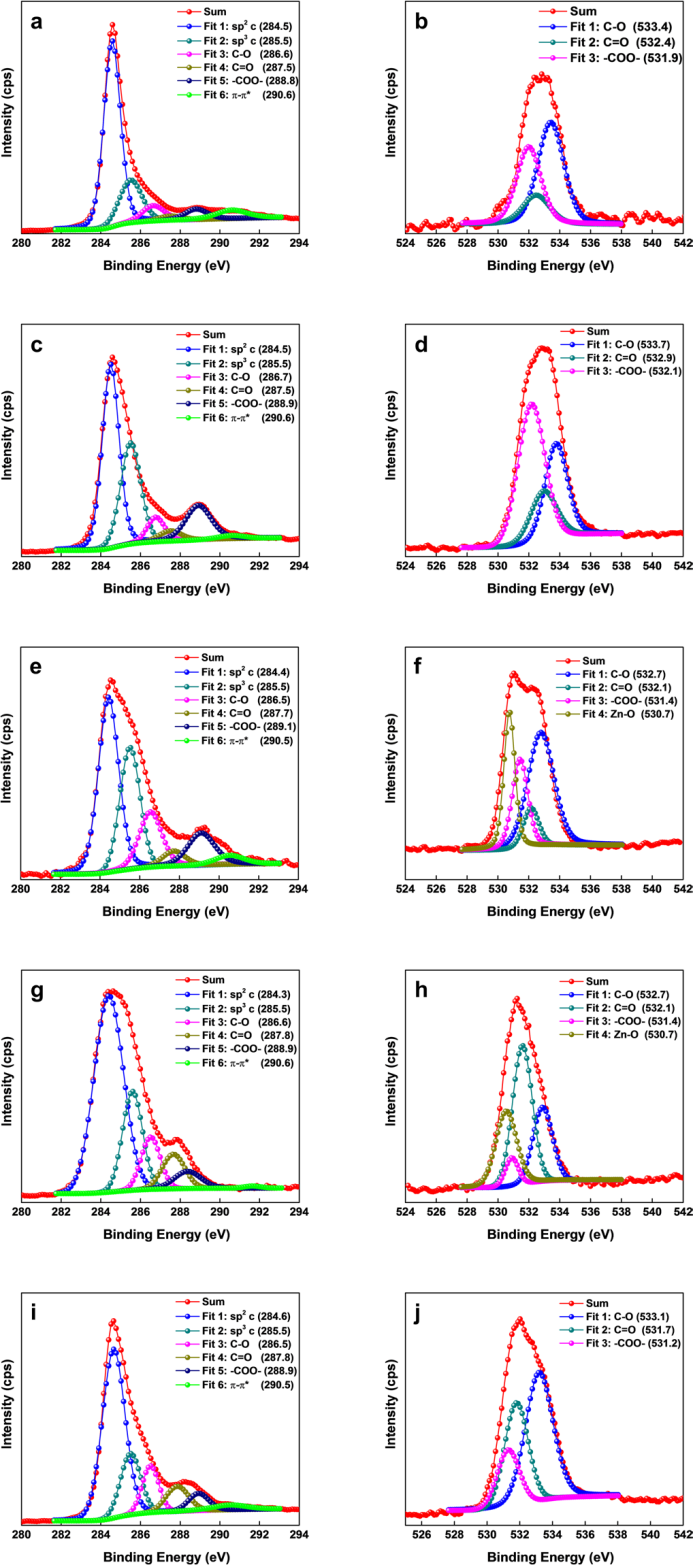
XPS core level spectra (**a**) C1s (**b**) O1s for neat SWCNTs (**c**) C1s (**d**) O1s for acid treated SWCNTs (**e**) C1s (**f**) O1s for ZnO-CNTs (**g**) C1s (**h**) O1s for ZnO-CNTs-PVP (**i**) C1s (**j**) O1s for NS-CNTs.

The X-ray photoelectron spectroscopy (XPS) spectra were measured at each processing step. Neat SWCNTs, acid-treated SWCNTs, ZnO-CNTs, ZnO-CNTs-PVP, and NS-CNTs were measured and compared. The presence of surface element can be detected through the XPS survey spectra (Fig. 5). It can be seen that neat SWCNTs consist of 93.27% carbon and 6.73% oxygen. After acid treatment, the content of the oxygen increases up to 22.07%. As acid treated SWCNTs reacted with zinc acetate dihydrate, zinc content starts to be shown to 29.85% and the oxygen content further increases up to 34.87%. When PVP is added, the carbon content increases again to 71.16% and the nitrogen content appears to be 5.89% coming from the composition of the PVP. Finally, after removal of core zinc atoms, all peaks related to zinc disappear and the surface element include 82.19% carbon, 3.68% nitrogen, and 14.13% oxygen. As shown in Table 1, due to the functionalization of SWCNTs, C/O ratio decreases after acid treatment of SWCNTs from 13.85to 3.53. But when NS-CNTs-PVP are synthesized, this effective C/O ratio of NS-CNTs without considering the contribution of PVP increases again up to 7.86, which is very close to that of neat SWCNTs. This means that intrinsic properties of SWCNTs are almost reverted because oxygen atoms participated in Zn-O-C bonding at the interface between core ZnO and outer SWCNTs are removed in the form of Zn-O by HCl and then only carbon atoms remain. The XPS spectra were taken into account to confirm both the change and chemical bonding of each component in the samples. Figure 6 illustrates the core-level C1s and O1s XPS spectra for each compound from the NS-CNTs synthesis step and Table 1 shows the amounts of carbon, nitrogen, oxygen, zinc and relative ratio of carbon and oxygen (C/O). The spectra were calibrated using C1s peak (284.6 eV).

**Table 1:** C, N, O, and Zn contents and relative C/O ratio (XPS) for each product.

Materials	C 1s [%]	N 1s [%]	O 1s [%]	Zn 2p^3/2^[%]	C/O
SWCNTs	93.27 (±0.19)	0.00	6.73 (±0.19)	—	13.85
Acid treated SWCNTs	77.93 (±0.19)	0.00	22.07 (±0.19)	—	3.53
ZnO-CNTs	35.28 (±0.59)	0.00	34.87 (±3.29)	29.85 (±3.88)	1.01
ZnO-CNTs-PVP	71.16 (±0.21)	5.89 (±0.23)	19.34 (±0.04)	3.61 (±0.02)	3.68
NS-CNTs-PVP	82.19 (±0.48)	3.68 (±0.77)	14.13 (±0.36)	—	5.81 (7.86)^a)^

^a)^Effective C/O ratio of NS-CNT without contribution of PVP.

**Figure 7:**
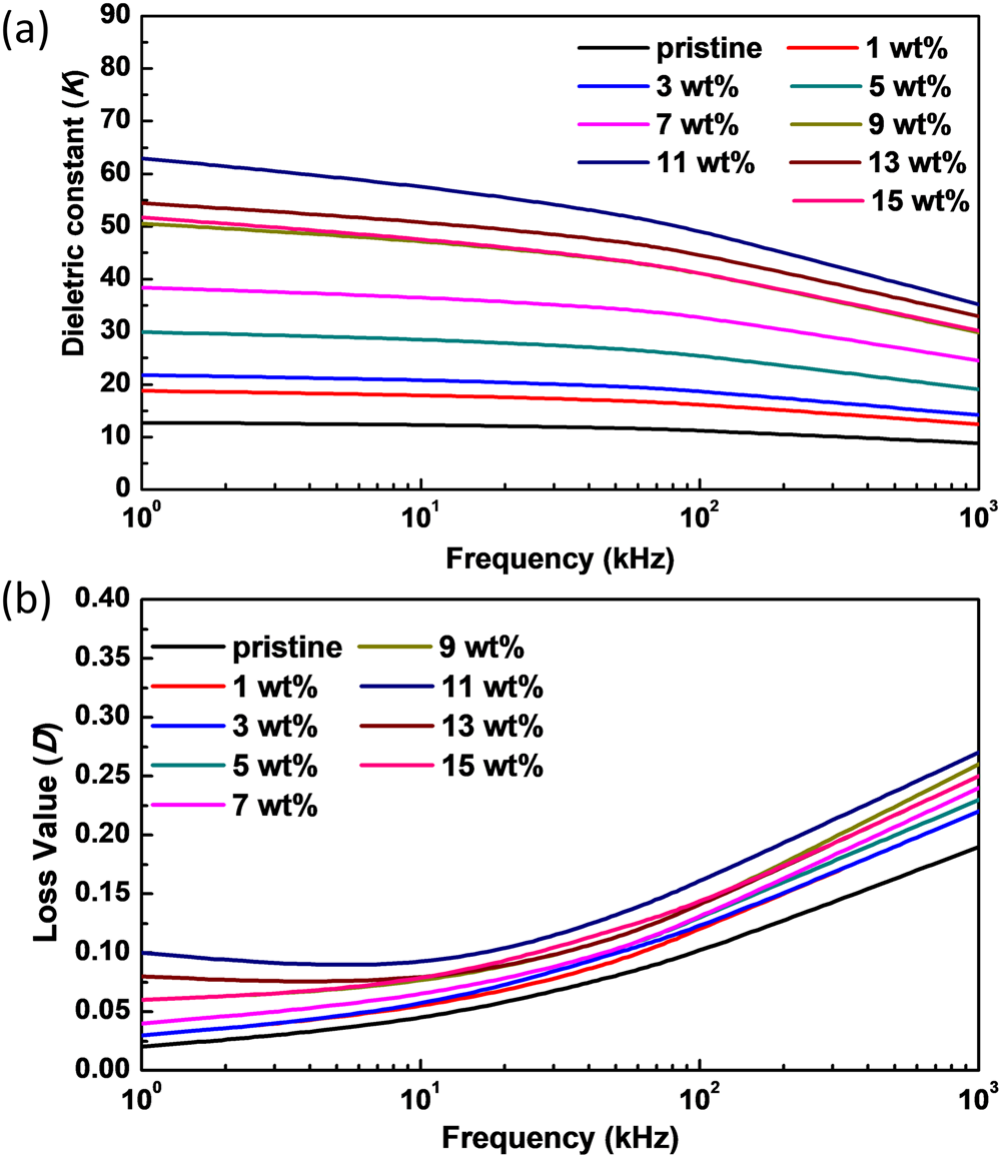
Measured dielectric constant data of the P(VDF-TrFE) and NS-CNTs-PVP nanocomposite: (**a**) and (**b**) shows variations of relative dielectric constant (*K*) and dielectric loss tangent (*D*) with frequency for NS-CNTs/P(VDF-TrFE) nanocomposites for various weight concentrations.

The C1s deconvoluted spectra show the changes in sp^2^ C, sp^3^ C, C-O, C=O, -COO-, and π-π* satellite peak and the O1s deconvoluted spectra show the changes of C-O, C=O, and -COO- bondings^30–32^. The first step of the chemical synthesis of NS-CNTs is the functionalization of as-procured SWCNTs. This process introduces hydroxyl, epoxy, carbonyl, and carboxyl groups on the surface of SWCNTs, which can be verified by the increase of C-O, C=O, and -COO- peaks in the XPS data of SWCNTs and acid-treated SWCNTs (Fig. 6a–d). Then, the ZnO-CNTs hybrid structures are formed. The increase in C-O peak and the formation of Zn-O peak is caused by the formation of C-O-Zn bonding through the chemical reaction between Zn(OH)_2_ and the functional groups on SWCNTs (Fig. 6e,f). When ZnO-CNTs are treated with PVP, the functional groups on the wall of SWCNTs create hydrogen bonding with PVP that causes the increase of sp^3^ C and C=O peaks (Fig. 6g,h). Finally, the XPS spectrum of NS-CNTs is similar to the spectrum of SWCNTs, but the slightly decreased sp^2^ C peak can be explained by the introduction of PVP wrapper (Fig. 6i,j). The synthesized NS-CNTs were then used for the fabrication of nanocomposite with P(VDF-TrFE) polymer (Figure S7). The weight content (wt%) of NS-CNTs in the nanocomposites varied from 1 to 15 wt%. The variation of dielectric constant and loss tangent as a function of frequency for all the nanocomposite self-supporting films is shown in Fig. 7a,b. The dielectric constant decreases and the loss value increases as the frequency varies from low to high. This behavior commonly observed in the dielectric constant of a polymer correlates with polarization rate. Dipole polarization cannot be exactly changed with the oscillation of applied electric field at high frequency. The pristine P(VDF-TrFE) film shows the relative dielectric constant (*K*) of 12.7 at 1 kHz, which is comparable to the value of 10~12 reported by the manufacturing company. As the relative weight content of buckled NS-CNTs-PVP varied from 1 to 15 wt%, the *K* (measured at 1 kHz) showed a gradual increase, eventually reaching to a maximum value of *K* = 62.5 for 11 wt% and then a slight decrease to *K* = 51.7 at 15 wt%. Such a gradual reduction in K at the amounts higher than 11 wt% will be closely related to an aggregation between filler particles. But this huge 492% increase of K (62.5) in P(VDF-TrFE) copolymer incorporated with CNTs have never been reported yet, except for those with metal or ceramic materials. However, the incorporation of buckled NS-CNTs-PVP beyond a critical 11 wt% seemed to deteriorate the dielectric property of P(VDF-TrFE). The dielectric loss tangent (*D*) measured on the NS-CNTs-PVP incorporated P(VDF-TrFE) nanocomposite showed a very low value of 0.1 at 1 kHz. As discussed in previous reports, the disadvantage of polymer composites with the metal particles has been the significant increase in dielectric loss tangent value due to their agglomeration^22,33^. In case of linear SWCNTs as fillers, more often than not they turn to be conducting beyond 1 wt% dispersion due to an increased cross linking and agglomeration^27,34,35^. In this regard, it is noteworthy that CNTs can be loaded up to 15% maintaining their dielectric nature. The reason for being improved dispersibility (see Figure S8) can be explained through the decrease of strong intermolecular van der Waals force between each filler particles. This decrease results from a high steric barrier being provided by PVP on the wall of CNTs^36^. In order to understand the huge increase in dielectric constant of composite film, by about 492% compared to the bare P(VDF-TrFE) film, we have calculated the permittivity with the theoretical models called Maxwell-Garnett equation, Bruggeman equation^37^ and self-consistent effective medium theory^38^. We have modeled the dielectric constant by varying the particle shape and the volume fraction of the interface as the number of NS-CNT layers increases. As a result, it’s found that the self-consistent effective medium theory (SC-EMT) fits well with the measured data when the dielectric constant of NS-CNTs is 600, and the dielectric Eshelby tensor which is related to the geometry and orientation of filler particle is 0.04. (More detailed modeling data is being prepared to be submitted as a subsequent paper). This data indicates that the conductivity of NS-CNTs is in the range of semiconducting materials and their shapes are close to oval.

From the dielectric results, when a conductive filler enters onto the ferroelectric polymer, an interfacial zone is formed between the filler and the ferroelectric polymer. It is well known that in a composite film having a large volume fraction of interfaces, the interfacial polarization is most likely to dominate. As a result, the dielectric constant increases due to the increased number of dipoles at the interfacial zone. CNTs having high electron density form many local capacitors with high charge density in the ferroelectric polymer, and that make the dielectric constant to increase. According to the minicapacitor principle^39^, the nanofillers loaded in a ferroelectric polymer matrix increases the dielectric constant by making numerous minicapacitors. For practical applications, what is required from a good capacitor is a high dielectric constant with low dielectric loss. Although, linear CNTs can increase the interfacial polarization due to the high surface area, but their high tendency to networking between them causes the high loss value. In the present work, the NS-CNTs as fillers not only show a large improvement in the dielectric constant, through interfacial polarization, of a composite but also with the low dielectric loss due to their high dispersion properties under 11 wt%.

## Conclusions

In conclusion, buckled and distorted hexagonal nanospring-shaped CNTs with a size smaller than 30 nm were synthesized by using ZnO NPs templates. The synthesized NS-CNTs were easily dispersed in P(VDF-TrFE) nanocomposites with an enhanced dielectric constant of about 492% at the content of 11 wt%. Since buckled NS-CNTs and P(VDF-TrFE) have no chemical bonding, the properties of nanocomposites can be closely related to the dispersibility of buckled NS-CNTs in P(VDF-TrFE) nanocomposites. Considering such a high dispersibility of NS-CNTs, NS-CNTs will be very useful as nanofillers for both nanofiber-type energy harvesting devices having few hundred nanometers of diameter and silicon based polymers commonly used for dielectric elastomer actuator (DEA)-based device requiring low operation voltage and low dielectric loss value^40,41^. Large increase of the dielectric constant of NS-CNTs-PVP incorporated P(VDF-TrFE) nanocomposite can be well explained by a nanocapacitor model.

## Methods

### Materials

Single-walled carbon nanotubes (SWCNTs) were purchased from Carbon Nanotech, Co., South Korea. (CNTs SP95, >95 wt%) Poly(vinylidene difluoride)-trifluoroethylene P(VDF-TrFE) 70/30 mol% copolymer powder used in this study was supplied by Piezotech S.A., France. The other chemicals obtained from Sigma-Aldrich Co. were used without further purification unless mentioned otherwise: zinc acetate dihydrate (≥98%); polyvinylpyrrolidone (PVP, Mw 40,000 g/mol); sodium dodecyl sulfate (SDS, ≥99%). The solvents, sulfuric acid (≥95%), nitric acid (70%), hydrochloric acid (37%), and dimethylformamide (DMF, 99%) were purchased and dried overnight over magnesium sulfate and filtrated using the 1μm pore size PTFE filter.

### Functionalization of SWCNTs

For the functionalization of SWCNTs, as-procured SWCNTs were dissolved in a mixture of sulfuric acid and nitric acid (H_2_SO_4_:HNO_3_ = 3:1). During this process, the SWCNTs were reported to have been cut into short tubes with most of the carboxylic groups (-COOH) covalently attached to the sidewalls, defects site, and open ends^42^. Using a multi-frequency ultrasonicator, the SWCNTs were dispersed in an acid mixture. After acid treatment, the solution was quenched in cold water, and the samples were diluted. Thereafter, the SWCNTs were extracted on a PTFE membrane of 1 um pore size using a vacuum filtration assembly. The resulting SWCNTs were dried in an oven at 70 °C overnight.

### Synthesis of ZnO-CNTs nanoparticles

A solution of zinc acetate dihydrate (1.84 g) in 300 ml dimethylformamide (DMF) was prepared under stirring, which formed Zn(OH)_2_. Acid-treated SWCNTs (20 mg) were dispersed in 50 ml DMF using a multi-frequency ultrasonicator and then added to the Zn(OH)_2_ solution under a continuous stirring for 5 hours at 150 °C. The solution was poured into ethanol. After the reaction was quenched, the resulting gray solid product was washed with ethanol and water, centrifuged, and then dried in the vacuum oven for overnight.

### Synthesis of PVP wrapped ZnO-CNTs

The ZnO-CNTs were dispersed in DI-water at a concentration of 50 mg/L with the aid of 1% SDS. 1% of the weight of the PVP was added to mixture. The solution was incubated at 50 °C for 12 hours. The products were then filtered through a 1 μm membrane filter, and washed with DI-water. This was followed by three cycles of ultrasonic redispersion in DI-water in order to remove residual SDS. Finally the sample was filtered and dried under vacuum at room temperature.

### Synthesis of buckled nano spring CNTs

ZnO-CNTs-PVP (100 mg) were dissolved in 30 mL hydrochloride acid (HCl), and subjected to sonication, and then poured into cold ethanol. After the reaction, the buckled NS-CNTs were washed with ethanol and water, centrifuged, and dried in a vacuum oven overnight.

### Fabrication of nanocomposite film

P(VDF-TrFE) was dissolved in DMF and the buckled NS-CNTs-PVP were dispersed in DMF, separately, by sonication for 1 hour. The P(VDF-TrFE) solution was then mixed with the buckled NS-CNTs-PVP solution. The mixed solution was mechanically stirred at room temperature for 1 hour and then sonicated for 1 hour. The solution was poured into a 4 cm × 7 cm template to make a free-standing film. The resulting composite was dried in a conventional oven for overnight. In order to measure the dielectric constant of the P(VDF-TrFE) composite with the variation of buckled NS-CNTs-PVP content, Au electrodes (100 nm) were deposited on the bottom and top sides by e-beam deposition.

### Characterization and Measurement

X-ray photoelectron spectroscopy (XPS) measurements were conducted with a ULVAC PHI5000 VERSA PROBE system with the Al Kα (1486.6 eV) X-ray source. The high resolution TEM measurements used JEM-4010 TEM operating at 400 keV. The TEM samples were prepared by dispersing sample dry powders in DMF to form a homogeneous suspension. The suspension was then dropped on a copper grid. The Raman spectra for the sample was recorded at room temperature by spreading the synthesized materials on a glass substrate and using a SPEX 1403 Laser Raman System with 785 nm argon ion laser excitation on a 300 lines/mm grating. The capacitance and dissipation factor were measured by using an Agilent 4284A LCR meter. TGA was measured with the SBTQ600 PA instrument increasing temperature 1°C/min in air.

## Electronic supplementary material

Supplementary Information
