# Supplementary material for: Highly Dispersible Buckled Nanospring Carbon Nanotubes for Polymer Nano Composites
Source: Sci Rep. 2018 Mar 19;8:4851. doi: 10.1038/s41598-018-23172-1 (PMC5859298; doi:10.1038/s41598-018-23172-1)
Supplement: Supplementary file 1 — Supplementary Information [file 41598_2018_23172_MOESM1_ESM.docx]

Supplementary Information

Highly Dispersible Buckled Nanospring CNTs for Polymer Nano Composites

Y.J. Lee, S.R. Ham, J.H. Kim, T.H. Yoo, S.R. Kim, Y.T. Lee, D.K Hwang, B. Angadi, W.S. Seo,

B.K. Ju^*^ and W.K. Choi^*^

**1. High resolution TEM image of the ZnO-CNTs**

Plane-view high resolution transmission electron microscopy (TEM) image of ZnO-CNTs is shown in Fig. S1. This HRTEM image clearly shows that the bundles of SWCNTs are surrounding the zinc oxide particle. The thickness of the SWCNTs is about 6.75 nm, which is equal to the diameter of the 8 turns of SWCNTs.


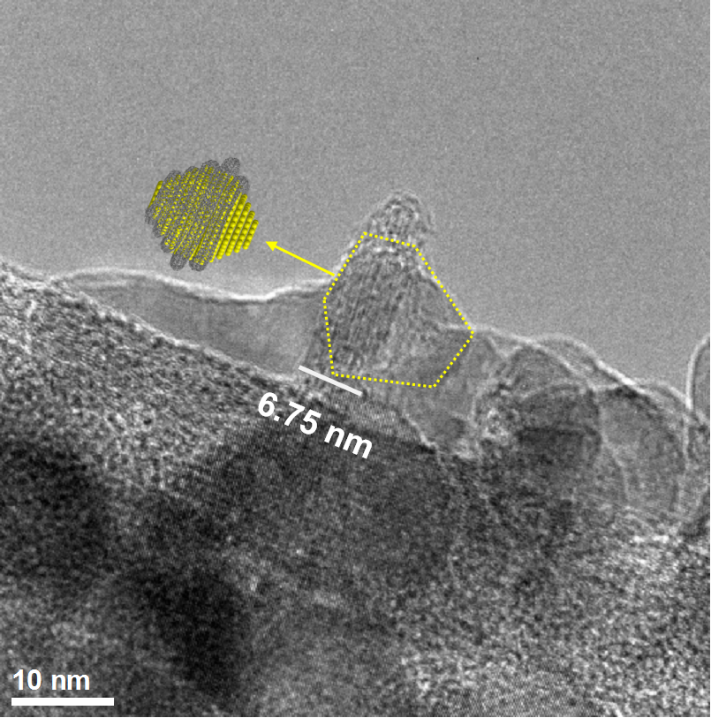


Figure S1. High resolution TEM image of the ZnO-CNTs structure. The length of 6.75 nm corresponds to the diameter of the eight turns of SWCNTs.

**2. X-ray diffraction patterns of ZnO-CNTs complex synthesized at various reaction time.**


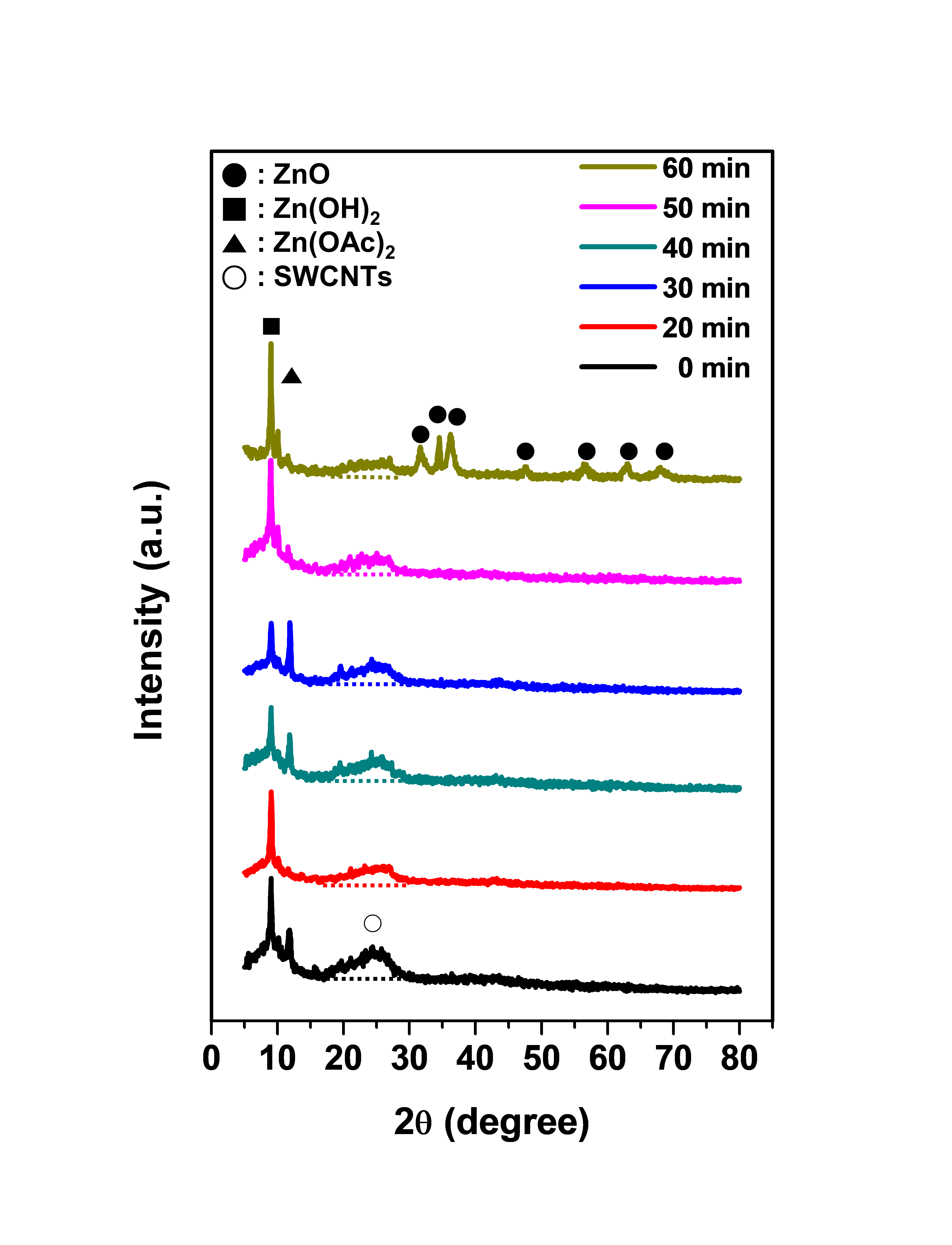


Figure S2. X-ray diffraction patterns of ZnO-CNTs complex synthesized at various reaction time.

**3. Thermogravimetric analysis (TGA) of ZnO-CNTs**

The TGA plot shows that the ZnO-CNTs sample loses water between 50 °C and 150 °C, which is 3.96 wt%. The weight loss of 3.18 wt% between 150 °C and 350 °C corresponds to a loss of hydroxyl group. Then, the sample loses an acetate anion between 350 °C and 500 °C, which is about 7.12 wt%. Finally, the SWCNTs start to decompose at 500 °C and do not exist after 800 °C due to complete decomposition. Since ZnO is stable up to 800 °C. The material which starts to decompose at 500 °C and ends at 800 °C is certainly believed as SWCNTs. It can be concluded that the content of SWCNTs in the ZnO-CNTs compound is about 4.84 %.


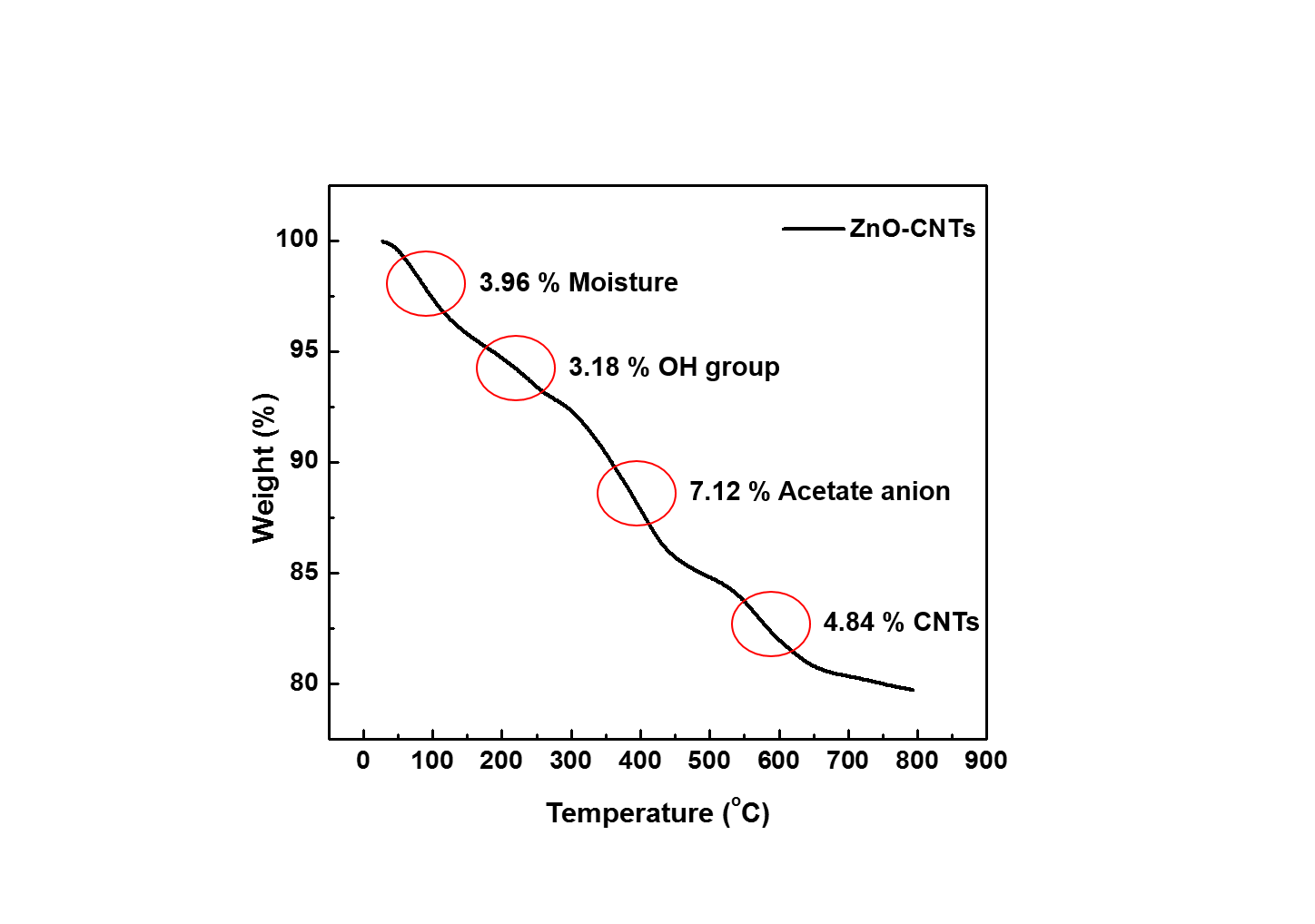


Figure S3. TGA data shows the weight loss of ZnO-CNTs of the range of 80 to 800 ^o^C.

**4. X-ray diffraction patterns of ZnO complex and various CNT materials.**


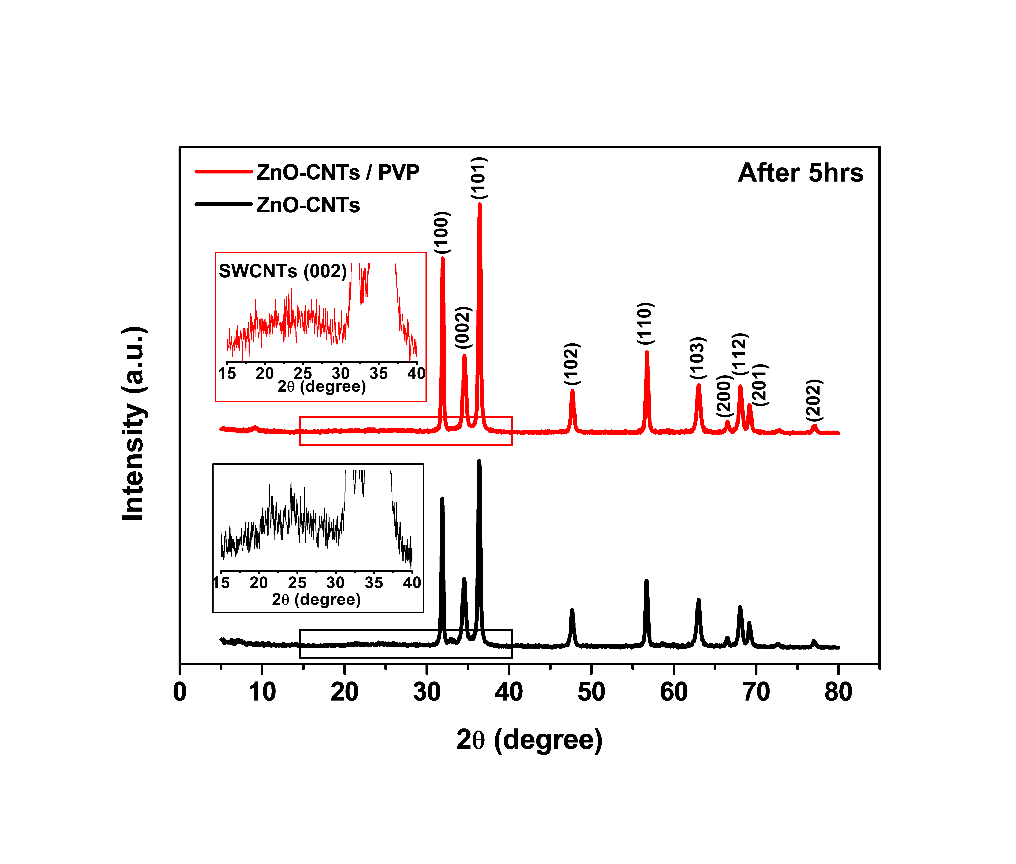

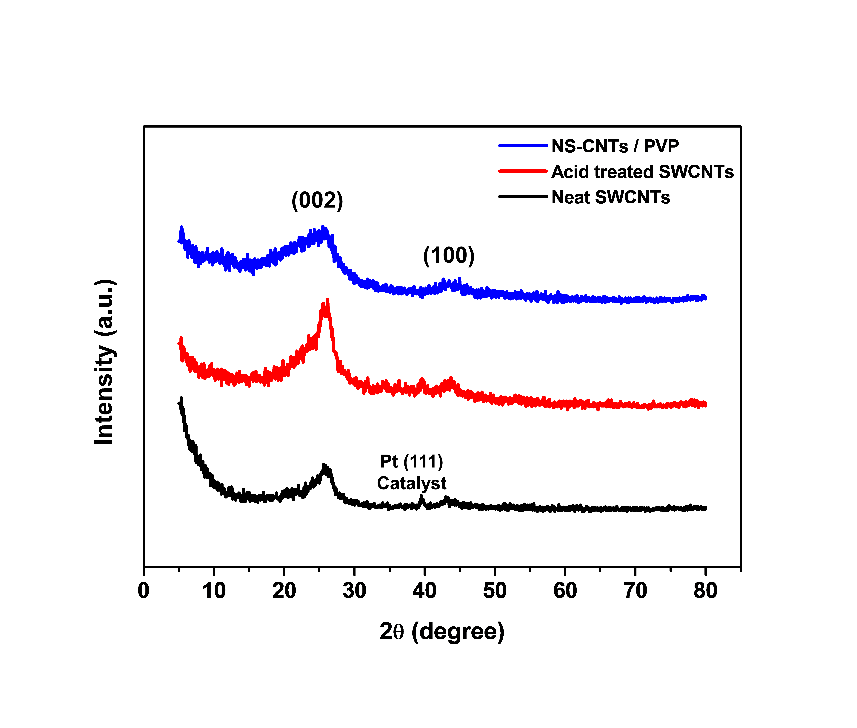


(b)

(a)

Figure S4. X-ray diffraction of the (a) ZnO-CNTs and ZnO-CNTs/PVP and (b) neat SWCNTs, acid treated SWCNTs and NS-CNTs/PVP.5. Thermogravimetric analysis (TGA) of PVP wrapped NS-CNTs

The sonicated SWCNTs blend and PVP were dissolved in water. In order for buckled SWCNTs to bind with PVP, each reagent was blended in solution using a multi frequency ultrasonicator. The TGA plot of NS-CNTs-PVP shows that, the synthesized sample loses some weight between 150 °C and 500 °C, followed by a steeper decrease of weight from 500-700 °C. The TGA data of the SWCNTs (black line in Fig. S5) shows that SWCNTs degrade at about 500 °C. The weight loss before reaching to 500 °C in the NS-CNTs-PVP TGA data (red line in Fig. S5) shows the degradation of PVP. Consequently, the relative weight content of PVP in buckled spring CNTs with PVP was analyzed to be 23 %.


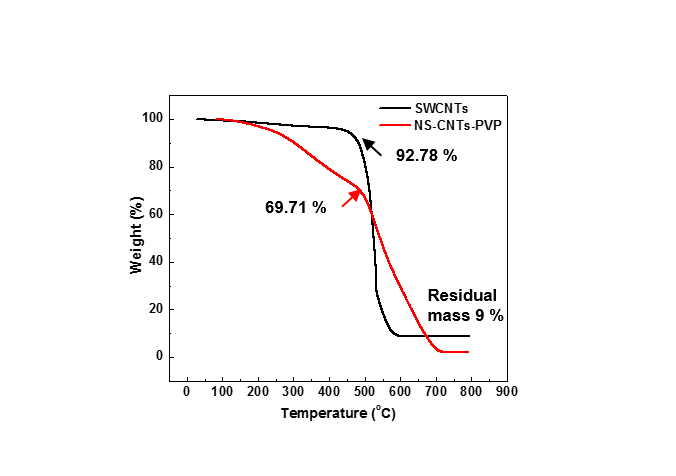


Figure S5. The TGA data shows the weight loss of SWCNTs (black) and NS-CNTs-PVP (red) in the range of 80 to 800 ^o^C.

**6. Raman spectroscopy analysis of neat SWCNTs, Acid treated SWCNTs, ZnO-CNTs, ZnO-CNTs/PVP and NS-CNTs/PVP.**

The relative ratio of integrated intensities of the D and G bands, I_D_/I_G_, can be used to estimate the density of defects in the SWCNTs structure: the larger is the value of the I_D_/I_G_ ratio, the higher is the defect density. As indicated in Figure S6, the value of I_D_/I_G_=0.89 for the acid treated SWCNTs is larger than that of 0.14 for neat SWCNTs. The values of I_D_/I_G_ are 0.52, 0.89, and 0.49 for ZnO-CNTs, ZnO-CNTs/PVP, and NS-CNTs respectively. The largest ratio of 0.89 are observed in both the acid treated SWCNTs and ZnO-CNTs/PVP.


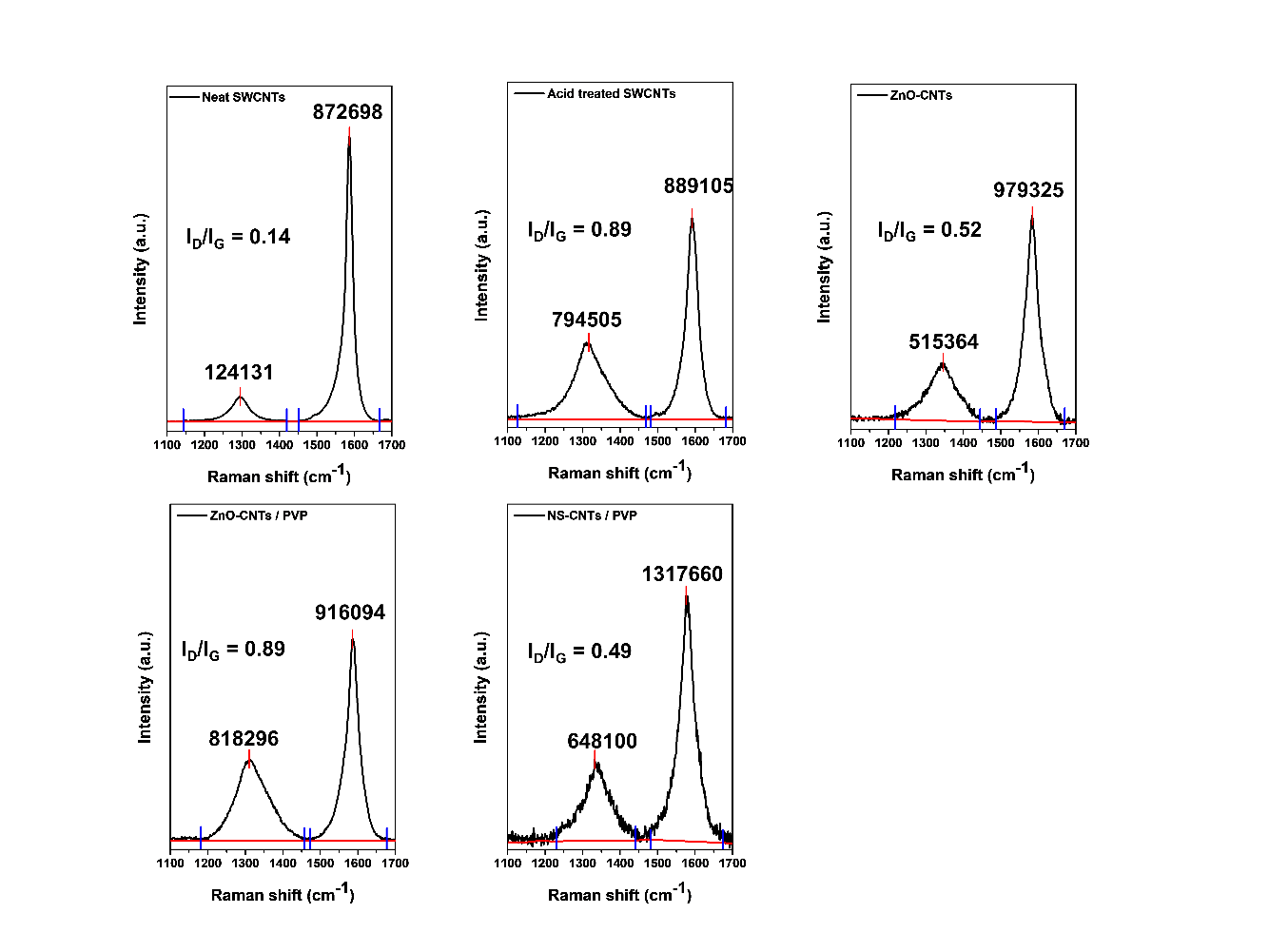


Figure S6. Raman shifts for neat SWCNTs, Acid treated SWCNTs, ZnO-CNTs, ZnO-CNTs/PVP and NS-CNTs/PVP and corresponding relative ratios of I_D_/I_G_. The red line is a baseline and blue bar is an integrated area.

7. Photographic image of P(VDF-TrFE) and NS-CNTs incorporated composite films

P(VDF-TrFE)-NS-CNTs nanocomposite films with several volume fractions of NS-CNTs contents (1-15 wt%) are fabricated through the ultrasonication mixing of solution and the free standing method. Images of P(VDF-TrFE) and NS-CNTs incorporated composite films are shown in Fig. S7. The dielectric constant of the nanocomposites was greatly enhanced from about 13 to a value higher than 60, for the contents of NS-CNTs were increased up to the value of 11 wt% while maintaining a low dielectric loss as the percolation threshold was formed.


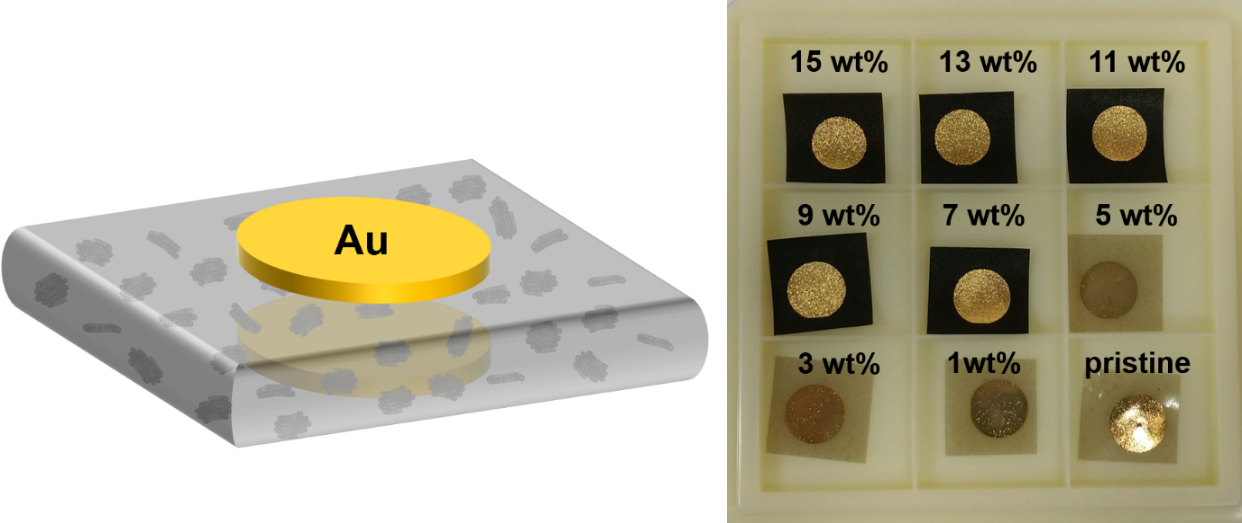


Figure. S7 Schematic image (left) and photographic image (right) of standing pristine P(VDF-TrFE) and NS-CNTs incorporated composite films with 60~80㎛ thickness.

**8. Photograph of neat SWCNTs and NS-CNTs dispersed in DMF**

Synthesized NS-CNTs showed excellent dispersibility. In compare to the poor dispersion state of neat SWCNTs, dispersed NS-CNTs in DMF have been kept even more than a month. Photographs of SWCNTs and NS-CNTs in DMF is shown in Fig. S8.


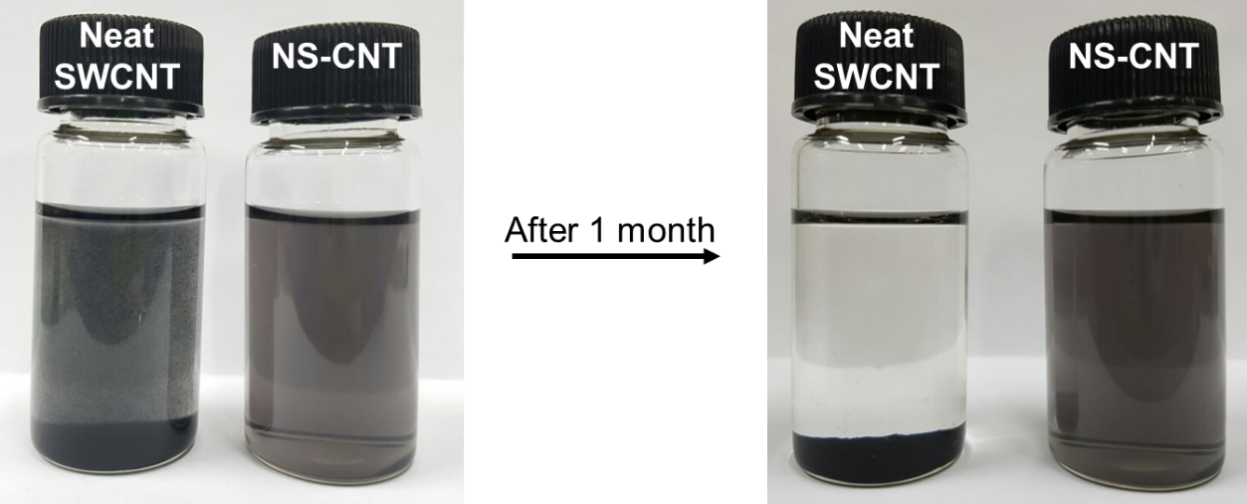


Figure S8. Photographs of neat SWCNTs and NS-CNTs dispersed in DMF.
